# Therapeutic inhibition of GAS6-AS1/YBX1/MYC axis suppresses cell propagation and disease progression of acute myeloid leukemia

**DOI:** 10.1186/s13046-021-02145-9

**Published:** 2021-11-09

**Authors:** Hao Zhou, Wei Liu, Yongming Zhou, Zhenya Hong, Jian Ni, Xiaoping Zhang, Ziping Li, Mengyuan Li, Wenjuan He, Donghua Zhang, Xuexing Chen, Jianhua Zhu

**Affiliations:** 1 Institute of Hematology, Union Hospital, Tongji Medical College, Huazhong University of Science and Technology, Wuhan, 430022 China; 2 Department of Hematology, The Affiliated Tianyou Hospital, Wuhan University of Science and Technology, Wuhan, 430064 China; 3 Department of Hematology, Tongji Hospital, Tongji Medical College, Huazhong University of Science and Technology, Wuhan, 430030 China; 4 Department of Oncology Clinical Pharmacy, Brigham and Women’s Hospital, Harvard Medical School, Boston, MA 02115 USA; 5 Department of Hematology, Xiangyang Central Hospital, Affiliated Hospital of Hubei University of Arts and Science, Xiangyang, 441000 China; 6 Laboratory of Clinical Immunology, Wuhan No. 1 Hospital, Tongji Medical College, Huazhong University of Science and Technology, Wuhan, 430022 China

**Keywords:** Oncogene, GAS6-AS1, MYC, YBX1, RNA-binding protein

## Abstract

**Background:**

Acute myeloid leukemia (AML) is the most common type of leukemia in adults. Its therapy has not significantly improved during the past four decades despite intense research efforts. New molecularly targeted therapies are in great need. The proto-oncogene c-Myc (MYC) is an attractive target due to its transactivation role in multiple signaling cascades. Deregulation of the MYC is considered one of a series of oncogenic events required for tumorigenesis. However, limited knowledge is available on which mechanism underlie MYC dysregulation and how long non-coding RNAs (lncRNAs) are involved in MYC dysregulation in AML.

**Methods:**

AML microarray chips and public datasets were screened to identify novel lncRNA GAS6-AS1 was dysregulated in AML. Gain or loss of functional leukemia cell models were produced, and in vitro and in vivo experiments were applied to demonstrate its leukemogenic phenotypes. Interactive network analyses were performed to define intrinsic mechanism.

**Results:**

We identified GAS6-AS1 was overexpressed in AML, and its aberrant function lead to more aggressive leukemia phenotypes and poorer survival outcomes. We revealed that GAS6-AS1 directly binds Y-box binding protein 1 (YBX1) to facilitate its interaction with MYC, leading to MYC transactivation and upregulation of IL1R1, RAB27B and other MYC target genes associated with leukemia progression. Further, lentiviral-based GAS6-AS1 silencing inhibited leukemia progression in vivo.

**Conclusions:**

Our findings revealed a previously unappreciated role of GAS6-AS1 as an oncogenic lncRNA in AML progression and prognostic prediction. Importantly, we demonstrated that therapeutic targeting of the GAS6-AS1/YBX1/MYC axis inhibits AML cellular propagation and disease progression. Our insight in lncRNA associated MYC-driven leukemogenesis may contribute to develop new anti-leukemia treatment strategies.

**Supplementary Information:**

The online version contains supplementary material available at 10.1186/s13046-021-02145-9.

## Background

Acute myeloid leukemia (AML) is a heterogeneous hematologic malignancy characterized by rapid cellular proliferation, aggressive clinical course and poor prognostic outcomes [1]. Since its therapy has not markedly improved during the past four decades despite intense research efforts, the ongoing research in the field is focused on developing new molecularly targeted therapies to improve the prognosis of AML [2, 3]. The proto-oncogene c-Myc (MYC) contributes to the genesis of many human cancers [4, 5]. *MYC* encodes a basic helix-loop-helix leucine zipper transcription factor that dimerizes with its partner, Max, and regulates multiple cellular functions including cell cycle, cell growth, differentiation, apoptosis, metabolism, and angiogenesis via transcription of downstream target genes [6, 7]. Recent insights into MYC expression and function have led to therapeutic opportunities in AML treatment [8, 9]. However, the mechanism underlying MYC dysregulation in the context of AML is obscure [10].

RNA-binding proteins (RBPs) are critical regulators of transcription and translation that are often dysregulated in cancer [11, 12]. Y-box binding protein 1 (YBX1), a member of the cold-shock protein superfamily that binds RNA to orchestrate transcription and translation, is a versatile RBP with a variety of interacting partners [13–15]. YBX1 is significantly upregulated in myeloid leukemia, and deletion of YBX1 dramatically induces apoptosis, promotes differentiation, and impaired leukemic capacity [16–18]. YBX-1 functions as a translational regulator and has been suggested to elevate MYC mRNA translation. Meanwhile, YBX-1 has the capacity to form an RNA nucleoprotein filament, cooperating with and stabilizing MYC [19, 20]. MYC is an attractive target for leukemia therapeutics due to its regulation by multiple signaling cascades, while the co-operator YBX-1 and its regulation of MYC activation is not well understood [21].

Recently, non-coding RNAs due to their involvement in vital oncogenic processes such as differentiation, proliferation, migration, angiogenesis and apoptosis have attracted much attention as potential diagnostic and prognostic biomarkers in leukemia [22, 23]. Our recent work revealed that non-coding RNA inhibits AML proliferation through selective RAB27B targeting [24]. Since long non-coding RNAs (lncRNAs) participate in transcriptional regulation within the cell and form regulatory networks [25–27], we sought to determine whether lncRNA was involved in MYC-driven leukemia proliferation and progression.

In this study, we found that GAS6 antisense RNA 1 (GAS6-AS1), a lncRNA, binds YBX1 to facilitate its interaction with MYC, resulting in transactivation of MYC and upregulation of IL1R1 and other known oncogenic transcripts from MYC, thereby performing leukemogenic activity. Preclinically, administration of lentivirus-mediated short hairpin RNA (shRNA) targeting GAS6-AS1 considerably suppresses acute myeloid leukemia cell propagation and disease progression, indicating the crucial roles of GAS6-AS1/YBX1/MYC axis in leukemia.

## Materials and methods

### Study subjects

From January 2014 to September 2015, bone marrow samples were collected from AML inpatients of Union Hospital, Tongji Medical College, Huazhong University of Science and Technology. Seventy-six patients had AML, including 41 males and 35 females, with an average age of 45.8 (17.3–76.6) years. The diagnostic type of AML was in accordance with the World Health Organization (WHO) classification criteria [28]. Clinical characteristics of the AML patients were listed in Table S1. Twenty bone marrow samples were obtained from healthy donors with a mean age of 38.7 years (ranging from 17.5 to 52.1) and a sex ratio of 11/9 (male/female).

### Bioinformatics analysis

Gene expression data were downloaded from the Cancer Genome Atlas (TCGA) and Gene Expression Omnibus (GEO) databases (GSE85030, GES103828, GSE37642 and GSE96535). The probe sequences were downloaded from GEO or microarray manufacturers, and bowtie was used to re-annotate probes according to GENCODE Release 19 annotation for lncRNAs.

### Cell culture

Human AML cell lines Kasumi-1, U937, MOLM-13, THP-1 and HL-60 were maintained in RPMI 1640 medium (HyClone, South Logan, UT) containing 10% fetal bovine serum (Gibco Cell Culture, Melbourne, Australia). HEK-293 T cells were maintained in RPMI DMEM medium (HyClone) containing 10% fetal bovine serum (Gibco). Kasumi-1, U937, MOLM-13, THP-1 and HL-60 cells were authenticated by karyotype, morphology, and PCR analysis. HEK-293 T cells were identified by morphology and capability of virus production.

### qRT-PCR

qRT-PCR was used to detect expression levels of GAS6-AS1 and other genes in AML cells, following the manufacturer’s instructions (TaKaRa, Japan). The β-actin was used as the control. Primers were listed in Table S2.

### Plasmid construction and cell transfection

The full-length cDNA of human GAS6-AS1, YBX1, MYC, and their truncations were synthesized by Invitrogen (Shanghai, China) and cloned into the lentiviral expression vector pWPXL. The small hairpin RNA (shRNA) constructs of GAS6-AS1, YBX1, and MYC were provided by GenePharma (Shanghai, China) and were cloned into the pLKO.1 shRNA lentiviral vector. For stable expression assays, all the vectors were packaged into lentiviruses with packaging plasmids psPAX2 and pMD2G in HEK-293 T cells. The resulting constructs were verified by sequence analysis. Finally, the lentiviruses were transfected into Kasumi-1, U937, and HL-60 cells, and the transformed cells were then selected with puromycin. All shRNA sequences were listed in Table S3.

### Cell proliferation and apoptosis assays

Cell proliferation ability was examined by CCK-8, colony formation, EdU and cell cycle assays. CCK-8 assay was conducted using a CCK-8 assay kit (Dojindo Japan). Ethynyl deoxyuridine (Edu) assay were conducted with EdU detection kit (KeyGEN BioTECH, Nanjing, China). Colony formation assays were performed according to standard protocols [29]. The FITC-Annexin V and propidium iodide (PI) double staining apoptosis assays were performed with flow cytometer (FACScan; BD Biosciences, USA).

### FISH and lncRNA distribution assay

The Cy3-labeled and FITC-labeled oligonucleotide probes were synthesized by Focobio Corporation (Guangzhou, China). Cells were incubated with FISH probes at 37 °C overnight. The nuclei were counterstained with DAPI. The slides were observed using fluorescent microscopy. For distribution assay, cytoplasmic and nuclear RNAs were isolated by the PARIS kit (Life Technologies, Carlsbad, CA) following the manufacturer’s protocols. The expression levels of GAS6-AS1 were measured by qRT-PCR.

### RNA pull-down and mass spectrometry

Biotin-labeled RNAs were in vitro transcribed using Biotin RNA Labeling Mix (Roche) and T7 RNA polymerase, treated with RNase-free DNase I, and purified with RNeasy Mini Kit (Qiagen). Nuclear extracts were harvested, resuspended in freshly prepared proteolysis buffer, and incubated with biotin-labeled RNA and streptavidin-agarose beads (Invitrogen). Precipitated components were separated using SDS-PAGE. Differential bands were harvested for mass spectrometry analysis.

### Western blot analysis

Western blot analysis according to standard protocols as described previously [29]. The antibodies including YBX1(ab76149, Abcam Inc.), MYC (ab32072), IL1R1 (ab106278), SRC (ab109381), RAB27B (ab76779), Histone H3 (ab18521), and β-actin (ab8226).

### RNA immunoprecipitation (RIP)

The EZ-Magna RIP Kit (Millipore) was used following the manufacturer’s protocol. Treated cells were lysed in complete RIP lysis buffer, and the cell extract was incubated with magnetic beads conjugated with specific antibodies or control IgG (Millipore). Beads were washed and incubated with Proteinase K to remove proteins. Finally, purified RNA was subjected to qRT-PCR analysis.

### Chromatin immunoprecipitation assays (ChIP)

ChIP experiments were performed using the Magna ChIP kit (Millipore) according to the manufacturer’s instructions. Real-time qPCR was undertaken with a SYBR Green PCR kit (TaKaRa) and primers targeting gene promoters were listed in Table S2.

### Co-immunoprecipitation (Co-IP)

Co-IP assay was conducted as described previously [30], with antibodies specific for YBX1 (ab76149) and MYC (ab32072). The bead-bound proteins were released and tested by Western blot.

### Dual-luciferase reporter assay

The promoter fragments of IL1R1 and RAB27B were amplified from genomic DNA by PCR and subcloned into pGL3-Basic (Promega). Primers were listed in Table S4. Dual-luciferase assay was performed according to manufacturer’s instruction (Promega). Luciferase reporters of MYC, EP300, TP53, and TFAP2A were obtained from Qiagen Inc. and Stratagene (La Jolla, CA). Luciferase activity was measured with a luminometer (Lumat LB9507, Berthold, Germany).

### RNA-seq assay

Total RNA of cells was extracted according to manual of TRIzol reagent (Life Technologies, Gaithersburg, MD). Library preparation and transcriptome sequencing was performed on an Illumina platform by Annoroad Gene Technology (Beijing, China). Sequencing data were deposited in SRA database under NCBI accession PRJNA737043.

### Animal experiment

Six- to eight-week old NOD-SCID mice were purchased from Beijing Vital River Laboratory Animal Technology (Beijing, China). The xenograft leukemia in mice was generated by injecting empty vector or sh-GAS6-AS1 transfected cells subcutaneously into a single side of each mouse. Three weeks after injection, xenografted mice were euthanized for analysis. For lentiviral based in vivo knockdown experiment, U937 cells were subcutaneously implanted into both sides of each mouse. Then, empty vector LeV-Scb and LeV-sh-GAS6-AS1 was intratumorally injected, twice a week for 2 weeks, at different side of each mouse. For systemic in vivo leukemia model, NOD-SCID mice were randomly divided in three groups and xenograft leukemia in mice was established by injecting 5 × 10^6^ sh-Scb or sh-GAS6-AS1 transduced U937 cells in 150 μL of phosphate-buffered saline (PBS) or PBS alone into the tail vein. Three weeks after inoculation, xenografted mice were euthanized for analysis. Human cell engraftment in bone marrow and spleen was examined by flow cytometry or hematoxylin and eosin (H&E) staining, as we have described previously [24]. The remaining mice were estimated using the survival analysis method.

### Positron emission tomography (PET) imaging

The mice were fasted and anesthetized on the 21st day after engraftment. Approximately 200 ± 20 μCi of 18-fluoro-6-deoxy-glucose (18F-FDG) was injected via the tail vein. Static PET images were started 1 h after the injection using a Trans-PET BioCaliburn LH system (Raycan Technology, Suzhou, China). A volume-of-interest analysis was conducted using the AMIDE software package (Free Software Foundation, Boston, USA).

### Statistical analysis

All results were depicted as mean ± standard error of the mean (s.e.m.). Student’s *t*-test, analysis of variance, and χ^2^ analysis were applied to compare difference. Fisher’s exact test was applied to analyze statistical significance of overlap between two gene lists. The survival curves are drawn using Kaplan–Meier survival plot and tested using log-rank tests. All statistical analyses were performed using SPSS 19 software (IBM, Somers, NY). *P* < 0.05 was considered to indicate statistical significance. Kaplan-Meier survival curves for mice and *P* values were calculated using a log-rank (Mantel-Cox) test.

## Results

### GAS6-AS1 is upregulated in AML and associated with poor prognosis

**Fig. 1:**
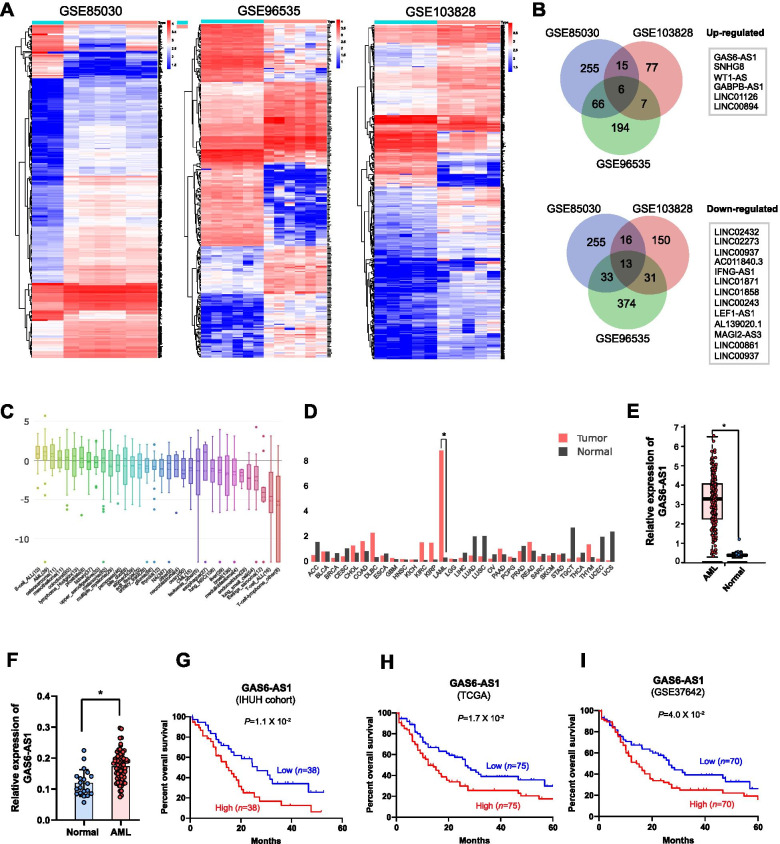
GAS6-AS1 is overexpressed in bone marrow of AML patients and AML cell lines, and is related to poor prognosis. **A** Hierarchical clustering analysis of differentially expressed lncRNAs (fold change > 2; *P* < 0.05) in AML and normal bone marrow mononuclear cells. **B** Overlap of misregulated lncRNAs in GEO datasets. **C** Analysis and comparison of GAS6-AS1 gene expression in cell lines of different tissue origins in the Cancer Cell Line Encyclopedia (CCLE) database. **D** Analyses of GAS6-AS1 expression levels in AML and other cancers using The Cancer Genome Atlas (TCGA) database. ACC, Adrenocortical carcinoma; BLCA, bladder urothelial carcinoma; BRCA, breast invasive carcinoma; CESC, cervical squamous cell carcinoma and endocervical adenocarcinoma; CHOL, cholangiocarcinoma; COAD, colon adenocarcinoma; DLBC, lymphoid neoplasm diffuse large B-cell lymphoma; ESCA, esophageal carcinoma; GBM, glioblastoma multiforme; HNSC, head and neck squamous cell carcinoma; KICH, kidney chromophobe; KIRC, kidney renal clear cell carcinoma; KIRP, kidney renal papillary cell carcinoma; LAML, acute myeloid leukemia; LGG, brain lower grade glioma; LIHC, liver hepatocellular carcinoma; LUAD, lung adenocarcinoma; LUSC, lung squamous cell carcinoma; MESO, mesothelioma; OV, ovarian serous cystadenocarcinoma; PAAD, pancreatic adenocarcinoma; PCPG, pheochromocytoma and paraganglioma; PRAD, prostate adenocarcinoma; READ, rectum adenocarcinoma; SARC, sarcoma; SKCM, skin cutaneous melanoma; STAD, Stomach adenocarcinoma; TGCT, testicular germ cell tumors; THCA, thyroid carcinoma; THYM, thymoma; UCEC, uterine corpus endometrial carcinoma; UCS, uterine carcinosarcoma; UVM, uveal melanoma. **E** Comparing the expression levels of GAS6-AS1 between AML and normal controls (TCGA vs GETx). **F** GAS6-AS1 expression was examined by qRT-PCR in AML (*n* = 78) and normal bone marrow mononuclear cells (*n* = 20). **G, H, I** Kaplan–Meier survival analysis of overall survival in AML patients, separately in three independent cohorts (IHUH, TCGA and GSE37642). Data were depicted as mean ± s.e.m., *P < 0.05

To identify the lncRNAs involved in leukemogenesis, we performed an integrative analysis of three gene expression profiles comprising GSE85030, GES103828, and GSE96535 datasets. All these datasets have enrolled bone marrow samples from both AML patients and normal persons. We identified 57 lncRNAs misregulated in both GSE96535 and GES103828 datasets, 118 in both GSE96535 and GSE85030 datasets, and 50 in both GSE85030 and GSE103828 datasets (fold change > 2.0, *P* < 0.05; Fig. 1A-B). Overlapping analysis revealed 6 lncRNAs upregulated and 13 downregulated in all datasets. Next, we focused on the most significant upregulated GAS6-AS1 as potential carcinogenic drivers or treatment targets. The Cancer Cell Line Encyclopedia (CCLE) data showed GAS6-AS1 expression was higher in AML cell lines than most of the other cancer types (Fig. 1C). We also studied the GAS6-AS1 expression by analyzing TCGA and Genotype-Tissue Expression (GTEx) database, as tumor and normal controls, respectively. The GAS6-AS1 expression was higher than all the other cancers (Fig. 1D). Additionally, comparing AML and normal controls (TCGA vs GETx), GAS6-AS1 was significantly upregulated in AML (Fig. 1E). Further, we examined GAS6-AS1 expression in an AML cohort in our Institute of Hematology of Union Hospital (IHUH), which revealed that GAS6-AS1 was significantly upregulated in AML patients and that 19.7% (15 of 76) patients showed more than 2-fold increase (Fig. 1F). Survival analysis showed that high GAS6-AS1 expression was significantly correlated with poor overall survival (Fig. 1G). Furthermore, we analyzed survival data from TCGA and GSE37642 and observed high GAS6-AS1 expression was associated with poor overall survival in AML (Fig. 1H-I). Collectively, these results indicated that GAS6-AS1 was overexpressed in AML, and it might contribute to leukemogenesis.

### GAS6-AS1 promotes AML cell propagation in vitro and disease progression in vivo

**Fig. 2:**
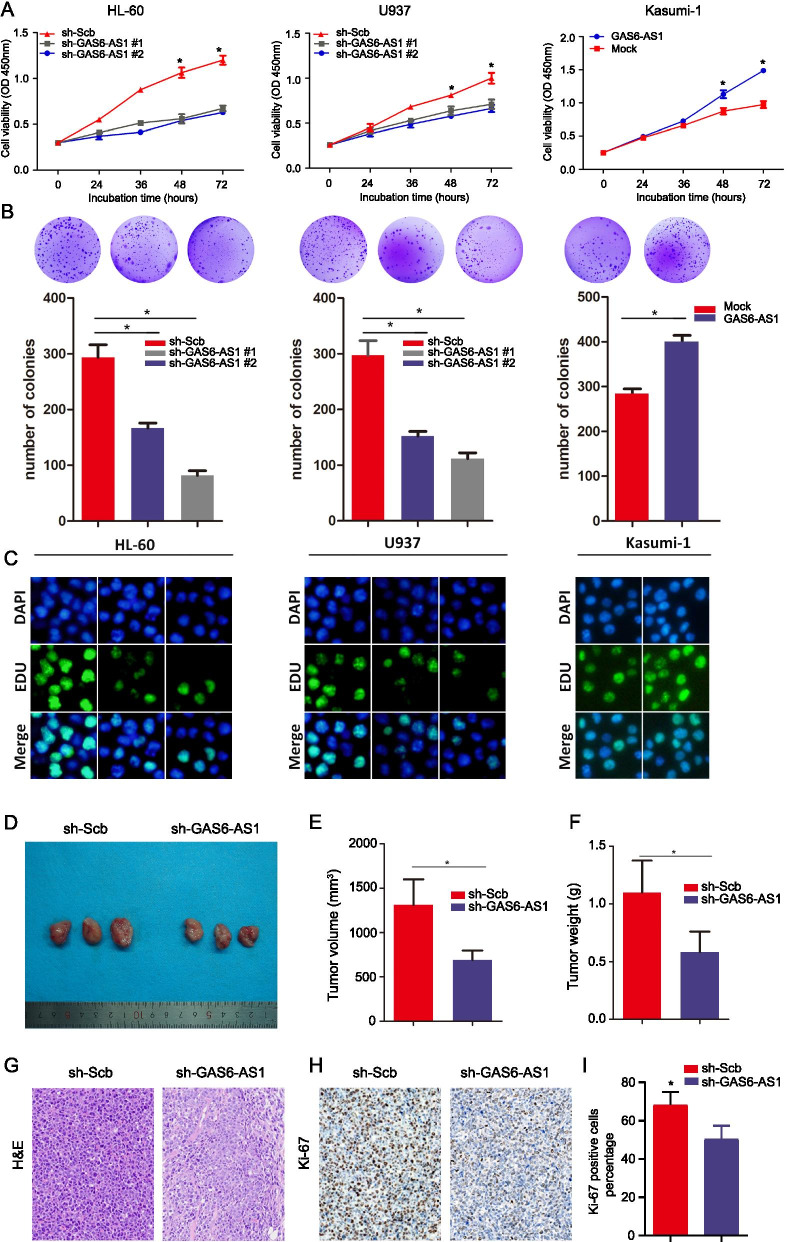
GAS6-AS1 promotes leukemia cell proliferation in vitro and vivo. **A** CCK-8 assays detecting cell proliferation of HL-60, U937 and Kasumi-1 cells following transfection-mediated GAS6-AS1 knockdown or overexpression. **B** Colony-forming assays detecting cell proliferation of HL-60, U937 and Kasumi-1 cells. **C** EdU assays examining cell proliferation after transfection. **D, E, F** Scramble vector or sh-GAS6-AS1 was transfected into U937 cells, which were subcutaneously injected in mice, and the tumors were obtained at day 21 and weighed (*n* = 5). **G, H, I** The tumor sections were under Hematoxylin and Eosin (H&E) staining and immunohistochemical staining using antibodies against Ki-67. Data were depicted as mean ± s.e.m., *P < 0.05

To assess the effect of GAS6-AS1 on AML cells, we tested the endogenous expression levels of GAS6-AS1 in various AML cell lines by qRT-PCR. The results revealed that compared with mononuclear cells (MNC) from bone marrow of healthy donors, the expression of GAS6-AS1 was significantly increased in U937, Kasumi-1, HL-60, MOLM-13 and THP-1 cell lines (*P* < 0.05), with U937 cell line exhibiting the highest level of GAS6-AS1 (Fig. S1). Accordingly, we chose U937 and HL-60 as loss of function experimental cell lines and Kasumi-1 as gain of function experimental cell line. Then EdU assays were performed and the results revealed that knockdown of GAS6-AS1 decreased HL-60 and U937 cells proliferation compared with the respective controls, whereas ectopic GAS6-AS1 overexpression promoted cell growth in Kasumi-1 (Fig. 2A). Colony-formation assays indicated that clonogenic survival was significantly declined following knockdown of GAS6-AS1 in HL-60 and U937 cells, but markedly increased in GAS6-AS1 overexpressed Kasumi-1 cells (Fig. 2B). Consistently, the EdU assay demonstrated that enforced expression of GAS6-AS1 had a positive impact on the leukemia cell proliferation (Fig. 2C). To strictly demonstrate the biological functions of GAS6-AS1 in different AML cell lines, we also conducted the cell viability, colony-formation and EdU assay in GAS6-AS1 overexpressed HL-60 and U937 cells and in GAS6-AS1 down-expressed Kasumi-1 cell. These results (supplementary data Fig. S2) were consistent with the results observed in GAS6-AS1 overexpressed Kasumi-1 cell and in GAS6-AS1 down-expressed HL-60 and U937 cells, respectively. To further investigate the disease progression effect of GAS61-AS1 in vivo, we subcutaneously inoculated NOD-SCID mice with xenograft leukemia tumor (Fig. 2D). Stable lentivector-based knockdown of GAS6-AS1 into U937 cells led to a significant augment in tumor weight, volume, and Ki-67 proliferative index of xenograft leukemia (Fig. 2E-I). Together, these results suggested the leukemogenic role of GAS6-AS1 in AML.

### GAS6-AS1 enhances AML cells proliferation by inducing cell-cycle progress and reducing apoptosis

**Fig. 3:**
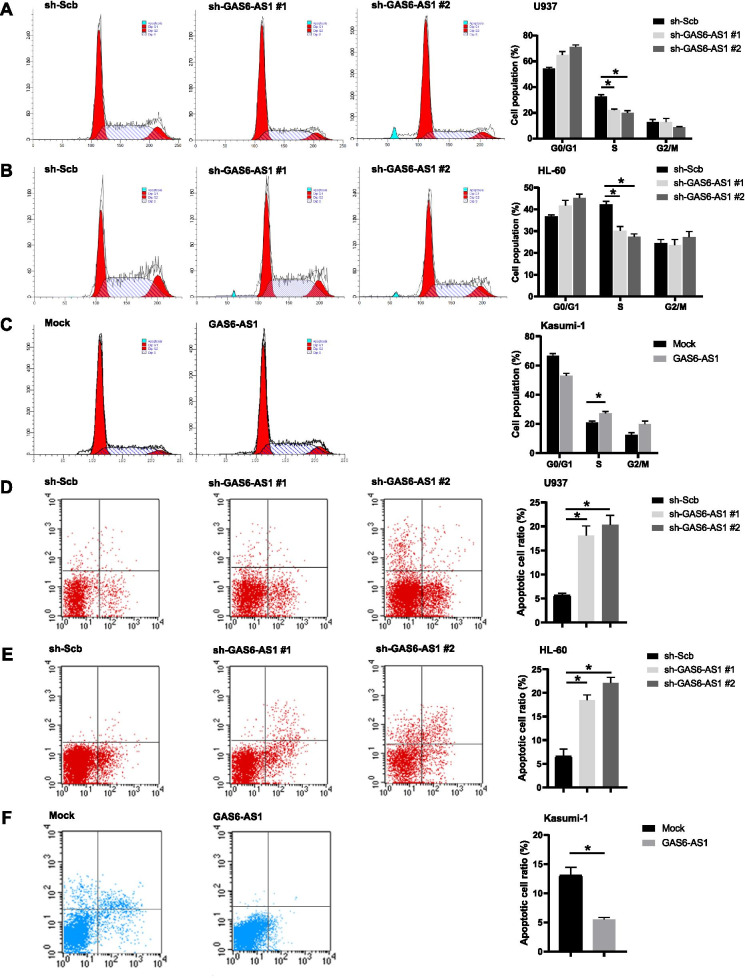
GAS6-AS1 enhances proliferation of leukemia cells by inducing G1-S cell cycle transition and reducing apoptosis. **A, B, C** The bar chart represented the percentage of U937, HL-60 and Kasumi-1 cells in G0/G1, S or G2/M phase, following transfection-mediated GAS6-AS1 knockdown or overexpression. **D, E** Flow cytometry detecting the apoptotic rates of U937, HL-60 and Kasumi-1 cells after GAS6-AS1 knockdown or overexpression. LR, early apoptotic cells; UR, terminal apoptotic cells. Data were depicted as mean ± s.e.m., *P < 0.05

To further explore whether GAS6-AS1 enhanced AML proliferation by regulation cell cycle progression in AML cells, flow cytometry was performed and demonstrated that HL-60 and U937 cells with sh-RNAs had a clear cell cycle arrest in the G1-S phase and the population of cells in the S phase was decreased (Fig. 3A-B). In Fig. 3A, the sh-GAS6-AS1#1 group showed no obvious apoptotic peak, while the sh-GAS6-AS1#1 group in Fig. 3D clearly showed apoptosis. We suspected that because the cell membrane of apoptotic cells is relatively fragile, it is possible that the apoptotic cells ruptured more severely during processing, resulting in most of them becoming invalid signals. This might lead to no obvious apoptotic peak in the sh-GAS6-AS1#1 group in Fig. 3A. However, forced expression of GAS6-AS1 induced G1-S progression and accumulated S phase (Fig. 3C). Apoptosis analysis indicated that the percentage of early and late apoptotic cells was significantly augmented in HL-60 and U937 cells with sh-GAS6-AS1 than the sh-Scb cells (Fig. 3D-E). Consistently, forced expression of GAS6-AS1 decreased apoptosis rate as compared with the empty vector group (Fig. 3F). These data revealed that GAS6-AS1 accelerated cell cycle progress and reduces apoptosis of AML cells.

### GAS6-AS1 directly interacts with YBX1 in AML cells

**Fig. 4:**
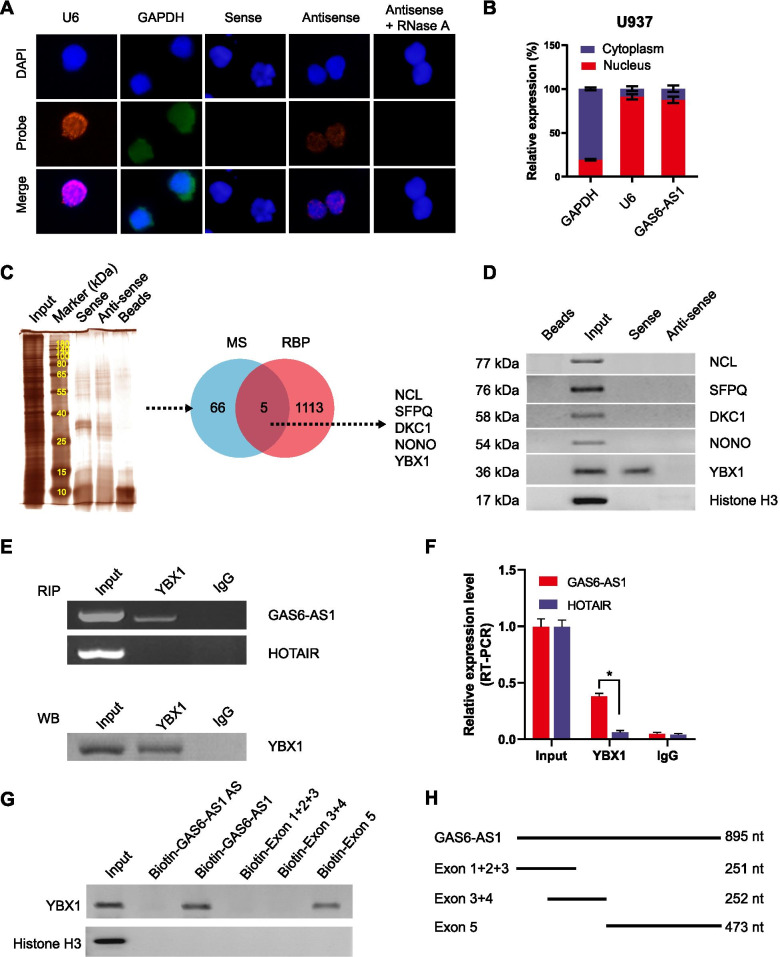
GAS6-AS1 directly interacts with YBX1 in AML cells. **A** RNA fluorescence in situ hybridization (FISH) using an antisense probe (red) revealing the localization of GAS6-AS1 in U937 cells. Sense probe and RNase A treatment were used as negative controls, while GAPDH (green) and U6 (red) were applied as cytoplasmic and nuclear controls, with nuclei staining by DAPI (blue). **B** Real-time qRT-PCR (normalized to β-actin, *n* = 4) showing the enrichment of GAS6-AS1 in the cytoplasm and nuclei of U937 cells. **C** Mass spectrometry (MS) of silver stained bands (left panel) and Venn diagram (right panel) indicating differential proteins pulled down by biotin-labeled GAS6-AS1 from nuclear extracts of U937 cells, and overlapping analysis with RNA-binding protein (RBP) database. **D** Biotin-labeled RNA pull-down and Western blot assays showing protein pulled down by GAS6-AS1 from lysates of U937 cells. The GAS6-AS1 antisense and bead-bound protein served as negative controls. **E-F** RIP and real-time qRT-PCR (normalized to input, *n* = 4) assays using YBX1 antibody indicating the interaction between GAS6-AS1 and YBX1 in U937 cells. The immunoglobulin G (IgG) and HOTAIR were applied as negative controls. **G** Biotin-labeled RNA pulldown assay revealing the interaction between GAS6-AS1 truncations and YBX1 protein in U937 cells. Biotin-labeled AS GAS6-AS1 served as a negative control. **H** Schematic illustration of the truncated vectors

To further functionally characterize GAS6-AS1, we analyzed the cellular distribution of GAS6-AS1 in U937 cells. As indicated by FISH assay and qRT-PCR, GAS6-AS1 was mainly located in the nucleus of AML cells (Fig. 4A-B; Fig. S3). We then screened potential GAS6-AS1-binding proteins via biotin-labeled RNA pull-down followed by mass spectrometry, which revealed 71 proteins pulled down by GAS6-AS1 in U937 cells (Fig. 4C; Table S5). Overlapping analysis with RNA-binding proteins defined by RBPDB (http://rbpdb.ccbr.utoronto.ca) revealed five potential partners of GAS6-AS1, including Nucleolin (NCL), Dyskerin Pseudouridine Synthase 1 (DKC1), YBX1, Splicing Factor Proline And Glutamine Rich (SFPQ), and Non-POU Domain Containing Octamer Binding (NONO). Further validating biotin-labeled RNA pull-down and Western blot assays demonstrated the direct interaction of GAS6-AS1 with YBX1, but not with NCL, DKC1, SFPQ or NONO (Fig. 4D). RIP assay confirmed the specific binding of YBX1 to GAS6-AS1, but not to the control primarily nuclear-retained lncRNA HOX transcript antisense RNA (HOTAIR), in U937 cells (Fig. 4E-F). Further catRAPID analysis revealed potential interaction region of GAS6-AS1 with YBX1 (Fig. S4). Meanwhile, the secondary structure of GAS6-AS1 was predicted by RNAfold webserver (Fig. S5). Thus, we constructed a series of truncated GAS6-AS1 to map its binding fragment with YBX1 (Fig. 4G-H). As verified by RNA pulldown assays, exon 5 of GAS6-AS1 was responsible for its interaction with YBX1 protein. These data indicated that GAS6-AS1 directly interacted with RNA binding protein YBX1 in AML cells.

### GAS6-AS1 increases MYC target gene expression through YBX1

**Fig. 5:**
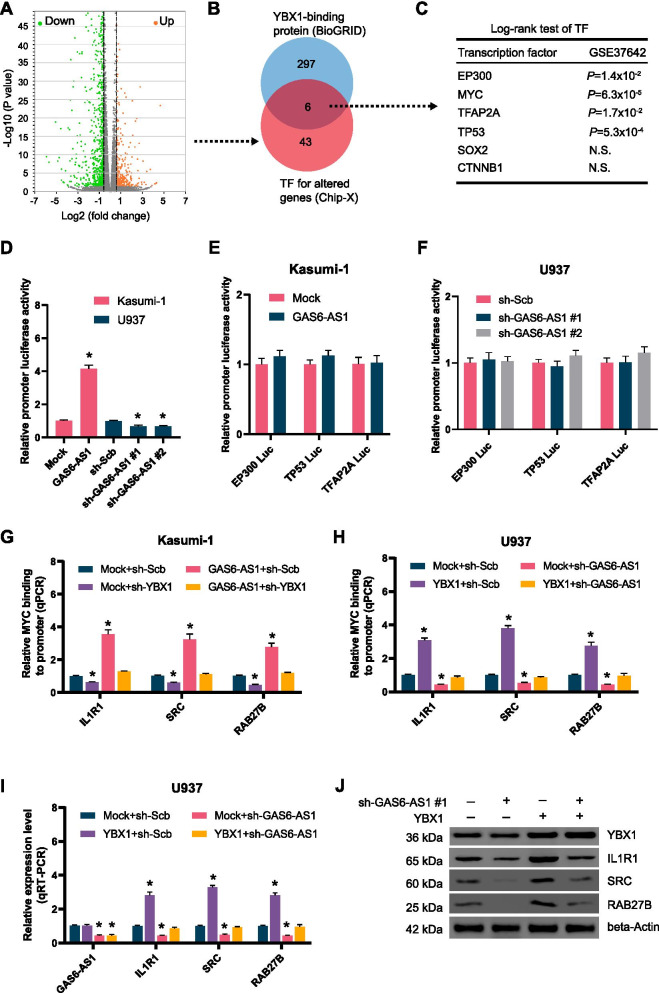
GAS6-AS1 increases MYC target gene expression through YBX1. **A** Volcano plots of RNA-seq showing the change of gene expression (fold change > 1.5, *P* < 0.05) in U937 cells stably transfected with scramble vector or GAS6-AS1 shRNA. **B** Venn diagram (left panel) was applied to identify transcription factors (TFs) regulating target gene expression, based on overlapping analysis of potential TFs using ChIP-X program and YBX1-interacting proteins from BioGRID database. **C** Log-rank test (right panel) showing the correlation of identified TFs with overall survival of AML cases (GSE37642). N.S., nonsignificant. **D** Dual-luciferase assay with a reporter carrying MYC binding sites showing the relative activity of MYC in Kasumi-1 and U937 cells stably transfected with empty vector (mock), GAS6-AS1, scramble shRNA (sh-Scb), or sh-GAS6-AS1 (*n* = 4). **E, F** Dual-luciferase assay indicating the relative activity of EP300, TP53, or TFAP2A in Kasumi-1 and U937 cells stably transfected with mock, MZF1-AS1, sh-Scb, or sh-MZF1-AS1 (*n* = 4). **G, H** ChIP and qPCR (normalized to input) indicating MYC enrichment at promoters of target genes in Kasumi-1 and U937 cells stably transfected with a series of vectors. **I** ChIP and real-time qRT-PCR (normalized to β-actin) showing the transcript levels of target genes. **J** Western blot showing protein levels of target genes in U937 cells stably transfected with sh-Scb, or sh-GAS6-AS1 #1, and those co-transfected with YBX1. Data were depicted as mean ± s.e.m., *P < 0.05

To identify the putative targets of GAS6-AS1, RNA sequencing (RNA-seq) assay revealed 155 upregulated and 348 downregulated genes (fold change > 1.5, *P* < 0.05) in U937 cells upon GAS6-AS1 knockdown (Fig. 5A). Further overlapping analysis of transcriptional regulators of these altered genes by ChIP-X program and YBX1-interacting protein in BioGRID database [31] revealed six potential transcription factors (Fig. 5B). Among these six transcription factors, the expression of E1A binding protein p300 (EP300), MYC, transcription factor AP-2α (TFAP2A), or tumor protein p53 (TP53) were reported to facilitate leukemogenesis and significantly associated with survival of AML (Fig. 5B). Notably, stable overexpression or knockdown of GAS6-AS1 modulated the activity of MYC, but not of EP300, TFAP2A, or TP53, in U937 and HL-60 cells (Fig. 5D-F). Among the MYC target genes derived from RNA-seq results and ChIP-X analysis, the expression of interleukin 1 receptor type I (IL1R1), proto-oncogene tyrosine-protein kinase src (SRC), and ras-related protein Rab-27B (RAB27B) was most significantly altered. Stably ectopic expression or silenced expression of GAS6-AS1 increased and decreased the MYC enrichment on target gene promoters in Kasumi-1 and U937 cells, which were rescued by silencing or forced expression of YBX1, respectively (Fig. 5G-H). Furthermore, alteration in expression of IL1R1, SRC and RAB27B induced by silencing of GAS6-AS1 was markedly rescued after forced expression of YBX1. (Fig. 5I-J). These findings implied that GAS6-AS1 enhanced the expression of MYC target genes through YBX1 in AML cells.

### GAS6-AS1 promotes propagation of AML cells via YBX1-mediated MYC transactivation

**Fig. 6:**
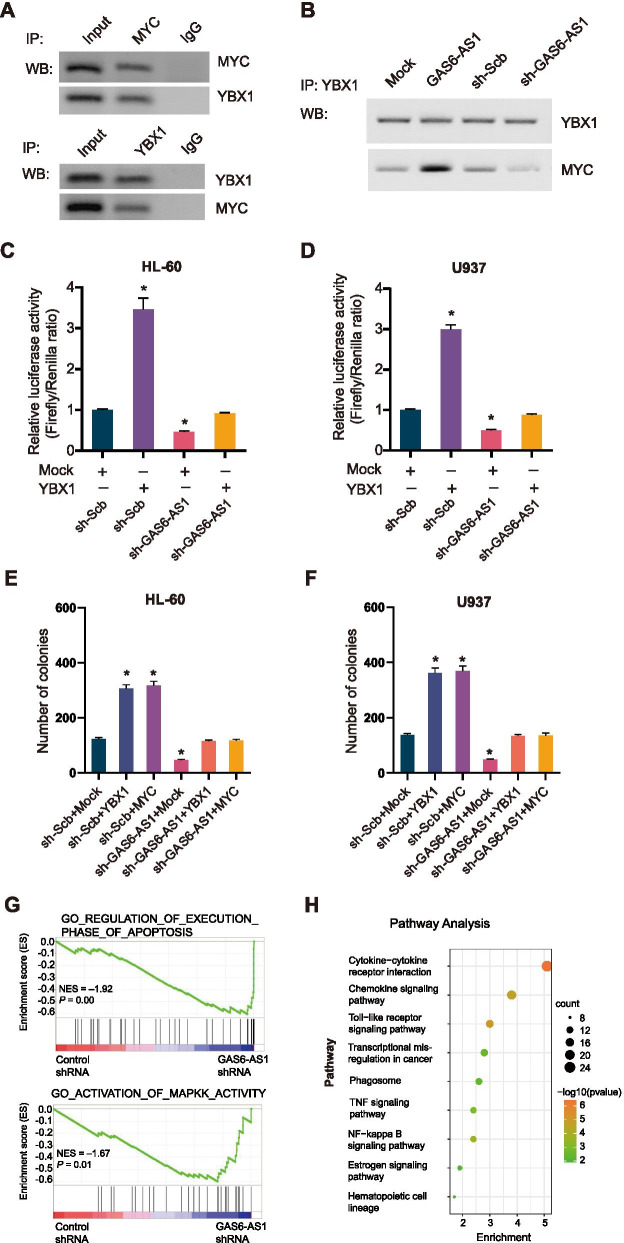
GAS6-AS1 facilitates leukemia cell proliferation via YBX1-mediated transactivation of MYC. **A** Co-IP and Western blot detecting endogenous interaction between YBX1 and MYC in U937 cells. IgG, negative control. **B** Co-IP and Western blot showing the interaction between YBX1 with MYC in U937 cells stably transfected with empty vector (mock), GAS6-AS1, scramble shRNA (sh-Scb), or sh-GAS6-AS1. **C, D** Dual-luciferase assay indicating the activity of MYC in AML cells stably transfected with sh-Scb, or sh-GAS6-AS1#1 and those co-transfected with YBX1 (*n* = 4). **E, F** Quantification of colony formation assay of leukemic cells transfected with a series of vectors. Data were depicted as mean ± s.e.m., *P < 0.05. **G** Gene Set Enrichment Analysis (GSEA) demonstrating the gene sets of regulation of apoptosis and the MAPK pathway were enriched in GAS6-AS1-downexpressing cells. **H** Pathway enrichment analysis using the Kyoto Encyclopedia of Genes and Genomes (KEGG)

Considering GAS6-AS1 bound to YBX1 and regulated MYC target genes, we hypothesized that GAS6-AS1 might act as a lncRNA linking YBX1 and MYC. Endogenous physical interaction between YBX1 and MYC was validated in U937 cells (Fig. 6A). Co-IP and Western blot assay demonstrated that knockdown of GAS6-AS1 suppressed the interaction between YBX1 and MYC (Fig. 6B). For the rescue experiments, both in HL-60 and U937 cells, forced overexpression of YBX1 alleviated the decreased MYC transactivation induced by silencing of GAS6-AS1 (Fig. 6C-D). Consistently, colony formation assay indicated the high proliferative phenotype of GAS6-AS1-silenced AML cells was rescued by overexpression YBX1 and MYC (Fig. 6E-F). In addition, Gene Set Enrichment Analysis (GSEA) demonstrated that the gene sets of regulation of apoptosis and the MAPK pathway were enriched in GAS6-AS1-silencing cells (Fig. 6G). The Kyoto Encyclopedia of Genes and Genomes (KEGG) pathway analysis suggested the potential implication of cytokine-receptor interaction (Fig. 6H). The GSEA and KEGG results were in accordance with reported main functions of those MYC target genes including IL1R1, SRC and RAB27B. Further, dual luciferase assays showed IL1R1 and RAB27B promoter activity was enhanced by MYC, while reduced by MYC knockdown (Fig. S6). These data indicated that GAS6-AS1 facilitated propagation of AML cells via YBX1-mediated MYC transactivation.

### Therapeutic knockdown of GAS6-AS1 inhibits AML disease progression

**Fig. 7:**
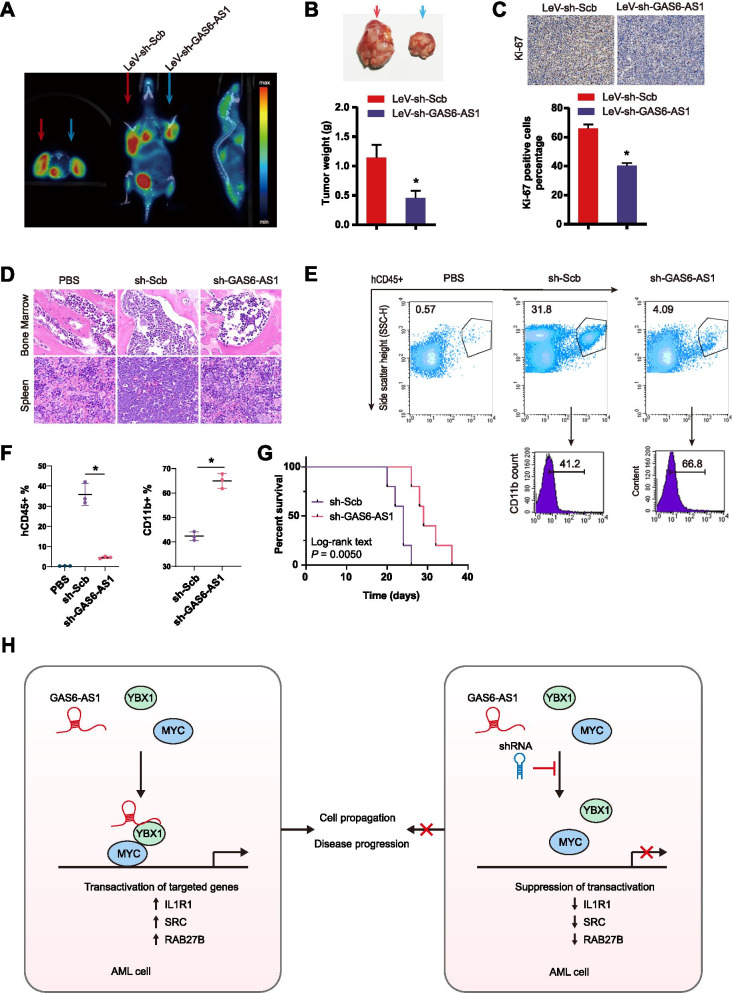
Therapeutically knocking-down GAS6-AS1 inhibits AML propagation and disease progression. **A** Representative PET images of tumor 18F-FDG uptake in leukemia-implanted mice after intra-tumoral injection of lentiviral vectors (*n* = 5 per group). **B, C** The weight and Ki-67 proliferation index in subcutaneous xenograft mice after lentiviral injection (red arrow: LeV-sh-Scb group; blue arrow: LeV-sh-GAS6-AS1 group). Data were depicted as mean ± s.e.m., *P < 0.05. **D** GAS6-AS1 knockdown impairs the tumorigenesis and infiltration of AML in vivo. H&E staining of bone morrow and spleen samples from mice xenotransplanted with U937 cells transfected with sh-Scb or sh-GAS6-AS1. **E** Flow cytometry showing declined levels of blasts in bone marrow from mice engrafted with GAS6-AS1-knockdown U937 cells compared with those in control group. CD11b marker expression was increased in sh-GAS6-AS1–treated group. **F** Scatter plots revealing the statistical values for panel **E**. **G** Kaplan-Meier survival curves for mice xenotransplanted with sh-Scb or sh-GAS6-AS1 U937 cells (*n* = 5 per group). *P* values were calculated using a log-rank (Mantel-Cox) test. **H** Schematic illustration of the mechanisms underlying GAS6-AS1 related oncogenic axis and therapeutic strategy

To analyze the therapeutic effect of lentivirus-mediated GAS6-AS1 knockdown on leukemic progression in vivo, subcutaneous tumorigenesis by U937 cells and thereafter scheduled lentiviral vectors injection were performed. The day of subcutaneous implantation was Day 0, and then the lentiviral vectors injection was conducted at Day 6, Day 10, Day 14, Day 18 (five subjects in each group). The PET scanning was performed at 21 days after engraftment. A decrease in the maximal standard uptake value of 18F-FDG was found in GAS6-AS1 shRNA-interfered group (Fig. 7A). Administration of lentivirus-mediated shRNA against GAS6-AS1 (LeV-sh-GAS6-AS1 #1) dramatically reduced the weight and Ki-67 proliferation index in subcutaneous xenograft mice (Fig. 7B-C). Additionally, GAS6-AS6 and downstream gene expression in the tumor tissue from LeV-sh-GAS6-AS1 group were dramatically decreased (Fig. S7). These data indicated that lentivirus based GAS6-AS1 knockdown inhibited leukemia progression.

In addition to the subcutaneous in vivo leukemia model, we also characterized the effect of GAS6-AS1 knockdown using systemic in vivo leukemia model. The NOD-SCID mice were injected via the tail vein with 5 X 10^6^ U937 cells with sh-GAS6-AS1 (knockdown) or sh-Scb (control). We assessed the organ infiltration meidated by GAS6-AS1 via H&E staining 3 weeks after injection. Compared with sh-Scb mice, sh-GAS6-AS1–treated mice exhibited reduced levels of U937 in the bone marrow and spleen, suggesting a lower degree of aggression in the indicated organs (Fig. 7D). Consistently, flow cytometry verified leukemia engraftment in the bone marrow and further demonstrated that sh-GAS6-AS1–treated mice exhibited lower percentages of hCD45^+^ cells in bone marrow than sh-Scb–treated mice (Fig. 7E-F). Remarkably, sh-GAS6-AS1 extended the survival of the recipients from 26 days for sh-Scb–treated mice to 36 days for sh-GAS6-AS1–treated mice (Fig. 7G; *P* = 0.0050), suggesting that GAS6-AS1 knockdown inhibits leukemia progression.

## Discussion

Understanding the tumor gene differential expression profiles can provide initial insights into how such changes may influence tumor development [32–34]. In this study, we compared gene expression profiles from AML patients with those from healthy persons by integrated bioinformatics analysis. We identified GAS6-AS1 as a significantly differential expressed gene, which was further proved to be an oncogenic lncRNA associated with poor prognosis. GAS6-AS1 promote the cooperation of YBX1 with MYC, leading to upregulation of downstream target genes associated leukemia progression, including IL1R1, SRC and RAB27B. The downstream genes IL1R1, SRC and RAB27B exert pivotal functions in GAS6-AS1-mediated leukemic cell propagation and disease progression (as schematically illustrated in Fig. 7H).

LncRNAs are over 200 nucleotides in length without protein-coding capacity, which play functional role through different mechanisms of action, according to their localization [22, 25]. Cytoplasmic lncRNAs participate to post-transcriptional regulation, mRNA turnover, protein stability and modulation of signaling pathways [35]. Instead, nuclear lncRNAs are mainly involved in chromatin remodeling, transcription regulation and nuclear architecture [36]. Through various mechanisms of action, lncRNAs participate to the regulation of a wide range of biological processes, such as cellular proliferation and differentiation, survival and apoptosis [37]. Notably, an important regulatory role of nuclear lncRNAs is modulation of transcription factors’ activities by cooperating with RNA-binding proteins, thereby effecting changes in target genes expression [38, 39]​. We identified that GAS6-AS1 was predominantly localized in the nucleus and our further evidence showed GAS6-AS1 could bind with certain RNA-binding proteins.

RNA-binding proteins are progressively considered as being critical for normal hematopoiesis and for hematological tumors as oncogenes [40–42]. YBX-1 is a multifunctional RNA-binding protein with an evolutionarily ancient and conserved cold shock domain, since its recognition Y-box element is present in the promoters of several genes associated with cell division [43]. It has also been shown that down-regulation of YBX1 results in reduced proliferation and increased apoptosis in myeloma cells [44]. More recently, our colleagues reported that YBX1 contributes to myeloid leukemia cell survival [17]. These data are in line with our observation that YBX1 is a vital cooperator of GAS6-AS1-associated pro-oncogenic genes transcription. Our results show that YBX1 bound to GAS6-AS1, and leukemia promoting functions of GAS6-AS1 were mediated, at least partly, through interacting with YBX1 in AML cells. Mechanistically, exon 5 domain of GAS6-AS1 interacted with YBX1 to enhance its interaction with MYC, resulting in transactivation of MYC. A lentiviral based shRNA vector targeting GAS6-AS1 was potent in suppressing leukemia cell proliferation and tumorigenesis, implying a potential therapeutic strategy for AML.

The Myc proteins are transcription factors with essential roles in cell growth and proliferation through their ability to transactivating gene expression [45–47]. Myc is frequently activated in AML and plays an important role in the induction of leukemogenesis [48, 49]. In particular, the MYC gene, located at 8q24, has been found to be one of the most commonly amplified regions in AML [50]. Myc is stabilized in AML leading to aberrant cytoplasmic localization of nucleophosmin (NPM), the most frequent genetic alteration in AML without karyotypic aberrations [51]. Transactivation by MYC appears to be mediated, at least partially, by accelerated RNA polymerase II elongation through interaction with certain cofactors [52], but it is unclear how this is regulated and which specific cofactors may be involved in controlling MYC-mediated transactivation. In this study, our findings indicated that YBX1 served as a co-factor of MYC in regulating target gene expression. Notably, our results revealed that GAS6-AS1 increased the transactivation of MYC, suggesting the crucial roles of MYC in GAS6-AS1-mediated downstream gene expression. To better observing the tumor development and therapeutic effect of intratumor injection by visual inspection and further by PET imaging, we chose subcutaneous in vivo leukemia model instead of intravenous model [53, 54]. Since systemic in vivo leukemia model is preferred for leukemic infiltration study [24, 55], apart from the subcutaneous in vivo tumor model, we also characterized the effect of GAS6-AS1 knockdown using xenotransplantation model. The results from systemic in vivo leukemia model were in constant with the in vivo tumor model, confirming that GAS6-AS1 knockdown impairs the tumorigenesis and infiltration of AML in vivo.

## Conclusions

Our study firstly identified that lncRNA GAS6-AS1 and its related GAS6-AS1/YBX1/MYC axis contribute to poor prognosis of AML patients, and exert oncogenic roles in regulating cellular proliferation and tumor progression. Mechanistically, GAS6-AS1 interacts with YBX1 to boost its interaction with MYC, leading to upregulation of IL1R1 and other oncogenic genes associated with leukemic progression [24, 56]. Lentiviral based knockdown of GAS6-AS1 exhibits a promising prospect in AML treatment. This study extends our knowledge about the regulation of cell proliferation and leukemic progression by lncRNA GAS6-AS1, and suggests that GAS6-AS1/YBX1/MYC axis may be a therapeutic target for AML. Further investigation is warranted to explore the roles of this axis in other types of tumors, and elucidate the functions and regulatory mechanisms of MYC and its oncogenic target gens in AML progression.

## Supplementary Information

**Additional file 1 **: **Table S1**. Clinical characteristics of the IHUH cohort. **Table S2**. Primer sets used for qRT-PCR, RIP, RT-PCR, probe, and ChIP. **Table S3**. The sequence of the shRNAs to diminish gene expression. **Table S4**. Primer pairs for luciferase vector construction. **Table S5**. Mass spectrometry analysis of proteins pulled down by GAS6-AS1. **Figure S1**. Real-time qRT-PCR (normalized to β-actin, *n* = 4) showing the expression of GAS6-AS1 in AML cell lines and mononuclear cells (MNC) from healthy donors. Data are shown as mean ± s.e.m. (error bars). *P < 0.05. **Figure S2**. GAS6-AS1 promotes leukemia cell proliferation in vitro and vivo. (A) CCK-8 assays detecting cell proliferation of HL-60, U937 and Kasumi-1 cells following transfection-mediated GAS6-AS1 overexpression or knockdown. (B-C) Colony-forming assays detecting cell proliferation of HL-60, U937 and Kasumi-1 cells. (D) EdU assays examining cell proliferation after transfection. Data were depicted as mean ± s.e.m., *P < 0.05. **Figure S3**. FISH analysis of GAS6-AS1 in HL-60 and Kasumi-1 cells using a biotin-labeled RNA probe. Nuclei were stained with DAPI. **Figure S4**. GAS6-AS1 physically interacts with YBX1 protein. The overall interaction propensity of GAS6-AS1 and YBX1 protein was predicted by catRAPID. **Figure S5**. The secondary structure of GAS6-AS1 predicted by RNAfold webserver. **Figure S6**. Dual luciferase reporter assays demonstrating the MYC enrichment and promoter activity of IL1R1 and RAB27B in AML cells stably transfected with sh-Scb, sh-MYC #1, sh-MYC #2, mock, or MYC (*n* = 4). Data are shown as mean ± s.e.m. (error bars). *P < 0.05. **Figure S7**. Real-time qRT-PCR (normalized to β-actin) indicating the expression of GAS6-AS1 and its target genes in xenograft tumors established by subcutaneous implantation of U937 cells into both dorsal flanks of each NOD-SCID mouse (*n* = 5 per group) that received intratumoral injection of LeV-sh-Scb or LeV-sh-GAS6-AS1 #1. Data are shown as mean ± s.e.m. (error bars). *P < 0.05.

## Data Availability

Our RNA-seq data used in this study (RNA-seq before and after GAS6-AS1 knockdown) have been deposited in SRA database under NCBI accession PRJNA737043. The lncRNA expression profiles data were obtained from GEO, with accession numbers GSE85030, GES103828, GSE37642 and GSE96535.

## Abbreviations

AML: Acute Myeloid Leukemia
CCLE: Cancer Cell Line Encyclopedia
ChIP: Chromatin Immunoprecipitation
Co-IP: Co-Immunoprecipitation
MYC: Proto-oncogene c-Myc
DKC1: Dyskerin Pseudouridine Synthase 1
EP300: E1A Binding Protein p300
NCL: Nucleolin
NONO: Non-POU Domain Containing Octamer Binding
PET: Positron Emission Tomography
qRT-PCR: Quantitative Reverse Transcription-polymerase Chain Reaction
RBP: RNA-binding Protein
RIP: RNA Immunoprecipitation
SFPQ: Splicing Factor Proline And Glutamine Rich
TFAP2A: Transcription Factor AP-2α
TCGA: The Cancer Genome Atlas
TP53: Tumor Protein p53
YBX1: Y-box Binding Protein 1

## References

[1] Döhner H, Weisdorf DJ, Bloomfield CD (2015). Acute myeloid leukemia. N Engl J Med.

[2] Winer ES, Stone RM (2019). Novel therapy in acute myeloid leukemia (AML): moving toward targeted approaches. Ther Adv Hematol.

[3] Blandino G (2021). Drugging the master regulator TP53 in Cancer: Mission possible?. J Clin Oncol.

[4] Chen H, Liu H, Qing G (2018). Targeting oncogenic Myc as a strategy for cancer treatment. Signal Transduct Target Ther.

[5] Gabay M, Li Y, Felsher DW (2014). MYC activation is a hallmark of cancer initiation and maintenance. Cold Spring Harbor Perspect Med.

[6] Scafuro M, Capasso L, Carafa V, Altucci L, Nebbioso A. Gene transactivation and Transrepression in MYC-driven cancers. Int J Mol Sci. 2021;22(7):3458. 10.3390/ijms22073458 PMC8037706 33801599

[7] Riddiough GE, Fifis T, Walsh KA, Muralidharan V, Christophi C, Tran BM, et al. Captopril, a renin-angiotensin system inhibitor, attenuates features of tumor invasion and down-regulates C-Myc expression in a mouse model of colorectal cancer liver metastasis. Cancers (Basel). 2021;13(11):2734. 10.3390/cancers13112734 PMC8199217 34073112

[8] de Oliveira LM, Brofman PRS, Schmid-Braz AT, Rangel-Pozzo A, Mai S. Chromosomal instability in acute myeloid leukemia. Cancers (Basel). 2021;13(11):2655. 10.3390/cancers13112655 PMC8198625 34071283

[9] Ohanian M, Rozovski U, Kanagal-Shamanna R, Abruzzo LV, Loghavi S, Kadia T (2019). MYC protein expression is an important prognostic factor in acute myeloid leukemia. Leuk Lymphoma.

[10] Reavie L, Buckley SM, Loizou E, Takeishi S, Aranda-Orgilles B, Ndiaye-Lobry D (2013). Regulation of c-Myc ubiquitination controls chronic myelogenous leukemia initiation and progression. Cancer Cell.

[11] Panzeri V, Manni I, Capone A, Naro C, Sacconi A, Di Agostino S (2021). The RNA-binding protein MEX3A is a prognostic factor and regulator of resistance to gemcitabine in pancreatic ductal adenocarcinoma. Mol Oncol.

[12] Pereira B, Billaud M, Almeida R (2017). RNA-binding proteins in cancer: old players and new actors. Trends Cancer.

[13] Gandhi M, Groß M, Holler JM, Coggins SAA, Patil N, Leupold JH (2020). The lncRNA lincNMR regulates nucleotide metabolism via a YBX1 - RRM2 axis in cancer. Nat Commun.

[14] Shurtleff MJ, Yao J, Qin Y, Nottingham RM, Temoche-Diaz MM, Schekman R (2017). Broad role for YBX1 in defining the small noncoding RNA composition of exosomes. Proc Natl Acad Sci.

[15] Pellanda P, Dalsass M, Filipuzzi M, Loffreda A, Verrecchia A, Castillo Cano V (2021). Integrated requirement of non-specific and sequence-specific DNA binding in Myc-driven transcription. EMBO J.

[16] Bhullar J, Sollars VE (2011). YBX1 expression and function in early hematopoiesis and leukemic cells. Immunogenetics..

[17] Feng M, Xie X, Han G, Zhang T, Li Y, Li Y, et al. YBX1 is required for maintaining myeloid leukemia cell survival by regulating BCL2 stability in an m6A-dependent manner. Blood. 2021;138(1):71–85. 10.1182/blood.2020009676 PMC8667054 33763698

[18] Liu S, Marneth AE, Alexe G, Walker SR, Gandler HI, Ye DQ (2018). The kinases IKBKE and TBK1 regulate MYC-dependent survival pathways through YB-1 in AML and are targets for therapy. Blood Adv.

[19] Cobbold LC, Wilson LA, Sawicka K, King HA, Kondrashov AV, Spriggs KA (2010). Upregulated c-myc expression in multiple myeloma by internal ribosome entry results from increased interactions with and expression of PTB-1 and YB-1. Oncogene..

[20] Bommert K, Effenberger M, Leich E, Küspert M, Murphy D, Langer C (2013). The feed-forward loop between YB-1 and MYC is essential for multiple myeloma cell survival. Leukemia..

[21] Wu Q-N, Luo X-J, Liu J, Lu Y-X, Wang Y, Qi J (2021). MYC-activated lncRNA MNX1-AS1 promotes the progression of colorectal cancer by stabilizing YB1. Cancer Res.

[22] Wang X, Li X, Lin F, Sun H, Lin Y, Wang Z (2021). The lnc-CTSLP8 upregulates CTSL1 as a competitive endogenous RNA and promotes ovarian cancer metastasis. J Exp Clin Cancer Res.

[23] Zhong C, Yu Q, Peng Y, Zhou S, Liu Z, Deng Y (2021). Novel LncRNA OXCT1-AS1 indicates poor prognosis and contributes to tumorigenesis by regulating miR-195/CDC25A axis in glioblastoma. J Exp Clin Cancer Res.

[24] Peng D, Wang H, Li L, Ma X, Chen Y, Zhou H (2018). miR-34c-5p promotes eradication of acute myeloid leukemia stem cells by inducing senescence through selective RAB27B targeting to inhibit exosome shedding. Leukemia..

[25] Tito C, Ganci F, Sacconi A, Masciarelli S, Fontemaggi G, Pulito C (2020). LINC00174 is a novel prognostic factor in thymic epithelial tumors involved in cell migration and lipid metabolism. Cell Death Dis.

[26] Verduci L, Tarcitano E, Strano S, Yarden Y, Blandino G (2021). CircRNAs: role in human diseases and potential use as biomarkers. Cell Death Dis.

[27] Turco C, Donzelli S, Fontemaggi G (2020). miR-15/107 microRNA Gene Group: characteristics and functional implications in cancer. Front Cell Dev Biol.

[28] Vardiman JW (2010). The World Health Organization (WHO) classification of tumors of the hematopoietic and lymphoid tissues: an overview with emphasis on the myeloid neoplasms. Chem Biol Interact.

[29] Chen L, Fan X, Zhu J, Chen X, Liu Y, Zhou H (2020). LncRNA MAGI2-AS3 inhibits the self-renewal of leukaemic stem cells by promoting TET2-dependent DNA demethylation of the LRIG1 promoter in acute myeloid leukaemia. RNA Biol.

[30] Yang F, Fang E, Mei H, Chen Y, Li H, Li D (2019). Acting promotes β-catenin signaling and cancer progression via DDX3-mediated transactivation of YY1. Cancer Res.

[31] Chatr-Aryamontri A, Oughtred R, Boucher L, Rust J, Chang C, Kolas NK (2017). The BioGRID interaction database: 2017 update. Nucleic Acids Res.

[32] Papaemmanuil E, Gerstung M, Bullinger L, Gaidzik VI, Paschka P, Roberts ND (2016). Genomic classification and prognosis in acute myeloid leukemia. N Engl J Med.

[33] Chung M, Bruno VM, Rasko DA, Cuomo CA, Muñoz JF, Livny J (2021). Best practices on the differential expression analysis of multi-species RNA-seq. Genome Biol.

[34] Sun Q, Guo D, Li S, Xu Y, Jiang M, Li Y, et al. Combining gene expression signature with clinical features for survival stratification of gastric cancer. Genomics. 2021;113(4):2683–94. 10.1016/j.ygeno.2021.06.018 34129933

[35] Yoon J-H, Abdelmohsen K, Gorospe M (2013). Posttranscriptional gene regulation by long noncoding RNA. J Mol Biol.

[36] Statello L, Guo C-J, Chen L-L, Huarte M (2021). Gene regulation by long non-coding RNAs and its biological functions. Nat Rev Mol Cell Biol.

[37] Peng W-X, Koirala P, Mo Y-Y (2017). LncRNA-mediated regulation of cell signaling in cancer. Oncogene..

[38] Zhang Q, Wei Y, Yan Z, Wu C, Chang Z, Zhu Y (2017). The characteristic landscape of lncRNAs classified by RBP–lncRNA interactions across 10 cancers. Mol BioSyst.

[39] Ferre F, Colantoni A, Helmer-Citterich M (2016). Revealing protein–lncRNA interaction. Brief Bioinform.

[40] Wang E, Lu SX, Pastore A, Chen X, Imig J, Chun-Wei Lee S (2019). Targeting an RNA-binding protein network in acute myeloid leukemia. Cancer Cell.

[41] Corley M, Burns MC, Yeo GW (2020). How RNA-binding proteins interact with RNA: molecules and mechanisms. Mol Cell.

[42] Hentze MW, Castello A, Schwarzl T, Preiss T (2018). A brave new world of RNA-binding proteins. Nat Rev Mol Cell Biol.

[43] Jurchott K, Bergmann S, Stein U, Walther W, Janz M, Manni I (2003). YB-1 as a cell cycle-regulated transcription factor facilitating cyclin a and cyclin B1 gene expression. J Biol Chem.

[44] Chatterjee M, Rancso C, Stühmer T, Eckstein N, Andrulis M, Gerecke C (2008). The Y-box binding protein YB-1 is associated with progressive disease and mediates survival and drug resistance in multiple myeloma. Blood..

[45] Dang CV (2012). MYC on the path to Cancer. Cell..

[46] Elliott B, Millena AC, Matyunina L, Zhang M, Zou J, Wang G (2019). Essential role of JunD in cell proliferation is mediated via MYC signaling in prostate cancer cells. Cancer Lett.

[47] Wolf E, Eilers M (2020). Targeting MYC proteins for tumor therapy. Annu Rev Cancer Biol.

[48] Kawagoe H, Kandilci A, Kranenburg TA, Grosveld GC (2007). Overexpression of N-Myc rapidly causes acute myeloid leukemia in mice. Cancer Res.

[49] L’abbate A, Tolomeo D, Cifola I, Severgnini M, Turchiano A, Augello B (2018). MYC-containing amplicons in acute myeloid leukemia: genomic structures, evolution, and transcriptional consequences. Leukemia..

[50] Salvatori B, Iosue I, Djodji Damas N, Mangiavacchi A, Chiaretti S, Messina M (2011). Critical role of c-Myc in acute myeloid leukemia involving direct regulation of miR-26a and histone methyltransferase EZH2. Genes Cancer.

[51] Bonetti P, Davoli T, Sironi C, Amati B, Pelicci PG, Colombo E (2008). Nucleophosmin and its AML-associated mutant regulate c-Myc turnover through Fbw7 gamma. J Cell Biol.

[52] Price DH (2010). Regulation of RNA polymerase II elongation by c-Myc. Cell..

[53] He X, Feng Z, Ma J, Zhang X, Ling S, Cao Y (2020). Bispecific and split CAR T cells targeting CD13 and TIM3 eradicate acute myeloid leukemia. Blood..

[54] Zheng X, Fan X, Fu B, Zheng M, Zhang A, Zhong K (2017). EpCAM inhibition sensitizes chemoresistant leukemia to immune surveillance. Cancer Res.

[55] Luo H, Zhu G, Xu J, Lai Q, Yan B, Guo Y (2019). HOTTIP lncRNA promotes hematopoietic stem cell self-renewal leading to AML-like disease in mice. Cancer Cell.

[56] Carey A, Edwards DK, Eide CA, Newell L, Traer E, Medeiros BC (2017). Identification of Interleukin-1 by functional screening as a key mediator of cellular expansion and disease progression in acute myeloid leukemia. Cell Rep.

