# Supplementary material for: Therapeutic inhibition of GAS6-AS1/YBX1/MYC axis suppresses cell propagation and disease progression of acute myeloid leukemia
Source: J Exp Clin Cancer Res. 2021 Nov 9;40:353. doi: 10.1186/s13046-021-02145-9 (PMC8576903; doi:10.1186/s13046-021-02145-9)
Supplement: Supplementary file 1 — Additional file 1 : Table S1. Clinical characteristics of the IHUH cohort. Table S2. Primer sets used for qRT-PCR, RIP, RT-PCR, probe, and ChIP. Table S3. The sequence of the shRNAs to diminish gene expression. Table S4. Primer pairs for luciferase vector construction. Table S5. Mass spectrometry analysis of proteins pulled down by GAS6-AS1. Figure S1. Real-time qRT-PCR (normalized to β-actin, n = 4) showing the expression of GAS6-AS1 in AML cell lines and mononuclear cells (MNC) from healthy donors. Data are shown as mean ± s.e.m. (error bars). *P < 0.05. Figure S2. GAS6-AS1 promotes leukemia cell proliferation in vitro and vivo. (A) CCK-8 assays detecting cell proliferation of HL-60, U937 and Kasumi-1 cells following transfection-mediated GAS6-AS1 overexpression or knockdown. (B-C) Colony-forming assays detecting cell proliferation of HL-60, U937 and Kasumi-1 cells. (D) EdU assays examining cell proliferation after transfection. Data were depicted as mean ± s.e.m., *P < 0.05. Figure S3. FISH analysis of GAS6-AS1 in HL-60 and Kasumi-1 cells using a biotin-labeled RNA probe. Nuclei were stained with DAPI. Figure S4. GAS6-AS1 physically interacts with YBX1 protein. The overall interaction propensity of GAS6-AS1 and YBX1 protein was predicted by catRAPID. Figure S5. The secondary structure of GAS6-AS1 predicted by RNAfold webserver. Figure S6. Dual luciferase reporter assays demonstrating the MYC enrichment and promoter activity of IL1R1 and RAB27B in AML cells stably transfected with sh-Scb, sh-MYC #1, sh-MYC #2, mock, or MYC (n = 4). Data are shown as mean ± s.e.m. (error bars). *P < 0.05. Figure S7. Real-time qRT-PCR (normalized to β-actin) indicating the expression of GAS6-AS1 and its target genes in xenograft tumors established by subcutaneous implantation of U937 cells into both dorsal flanks of each NOD-SCID mouse (n = 5 per group) that received intratumoral injection of LeV-sh-Scb or LeV-sh-GAS6-AS1 #1. Data are shown as mean ± s.e.m. (error bars). *P < 0.05. [file 13046_2021_2145_MOESM1_ESM.pdf]

Table S1. Clinical characteristics of the IHUH cohort.

Variable	N (%)
**Gender**	
Male	41 (53.9%)
Female	35 (46.1%)
**Age**	
≤60	52 (68.4%)
>60	24 (31.6%)
**Subtype**	
M0	4 (5.3%)
M1	20 (26.3%)
M2	26 (34.2%)
M4	16 (21.1%)
M5	7 (9.2%)
M6	3 (3.9%)
M7	0 (0.0%)

IHUH, Institute of Hematology of Union Hospital.

Table S2. Primer sets used for qRT-PCR, RIP, RT-PCR, probe, and ChIP

Primer set	Primers	Sequence	Application	Product length (bp)
GAS6-AS1	Forward	5'-CTTGCTCCTGCCGTGTAAGA-3'	qRT-PCR, RIP	160
	Reverse	5'-TGTCACACCGGTAGGAATGC-3'		
GAS6-AS1	Forward	5'-GCTCCTGCCGTGTAAGAAGT-3'	Probe	154
	Reverse	5'-CACACCGGTAGGAATGCAGT-3'		
GAPDH	Forward	5'-GGGCTGCTTTTAACTCTGGT-3'	qRT-PCR, Probe	198
	Reverse	5'-TGATTTTGGAGGGATCTCGC-3'		
U6	Forward	5'-AAAGCAAATCATCGGACGACC-3'	qRT-PCR, Probe	181
	Reverse	5'-GTACAACACATTGTTTCCTCGGA-3'		
HOTAIR	Forward	5'-AACTCTGACTCGCCTGTGCTCT-3'	RT-PCR	233
	Reverse	5'-CTTCTAAATCCGTTCCATTCCA-3'		
IL1R1	Forward	5'-ATGAAATTGATGTTCGTCCCTGT-3'	qRT-PCR	184
	Reverse	5'-ACCACGCAATAGTAATGTCCTG-3'		
SRC	Forward	5'-TTTGGCAAGATCACTAGACGGG-3'	qRT-PCR	111
	Reverse	5'-GAGGCAGTAGGCACCTTTTGT-3'		
RAB27B	Forward	5'-TAGACTTTCGGGAAAAACGTGTG-3'	qRT-PCR	192
	Reverse	5'-AGAAGCTCTGTTGACTGGTGA-3'		
MYC	Forward	5'-GTCAAGAGGCGAACACACAAC-3'	qRT-PCR	162
	Reverse	5'-TTGGACGGACAGGATGTATGC-3'		
YBX1	Forward	5'-GGGGACAAGAAGGTCATCGC-3'	qRT-PCR	155
	Reverse	5'-CGAAGGTACTTCCTGGGGTTA-3'		
β-actin	Forward	5'-TGCCCATCTACGAGGGGTATG-3'	qRT-PCR	156
	Reverse	5'-TCTCCTTAATGTCACGCACGATTT-3'		
IL1R1	Forward	5'-TATTTCTCAAGTTACCCAGGCACA-3'	ChIP	162
	Reverse	5'-GGAGCCGTCAATGAAGTTTT-3'		
SRC	Forward	5'-CAGGCCAGGTGGTGGTTAGG-3'	ChIP	155
	Reverse	5'-CCCAACCCACCCACCTTCTAC-3'		
RAB27B	Forward	5'-GTTGGGACTTGCAGACACGC-3'	ChIP	174
	Reverse	5'-GCTACTTATCTCCTCCAACCTGTG-3'		

Table S3. The sequence of the shRNAs to diminish gene expression.

sh-RNA	Sequence
sh-GAS6-AS1 #1 F	5'-CCGGGCCTGAACTAGAGAAATGATTCTCGAGAATCATTTCTCTAG TTCAGGCTTTTTG-3'
sh-GAS6-AS1 #1 R	5'-AATTCAAAAAGCCTGAACTAGAGAAATGATTCTCGAGTCATTTCT CTAGTCAGGCTT-3'
sh-GAS6-AS1 #2 F	5'-CCGGCTGGCCTGAACTAGAGAAATTCTCGAGTTTCTCTAGTTCAG GCCAGTTTTTTTG-3'
sh-GAS6-AS1 #2 R	5'-AATTCAAAAACTGGCCTGAACTAGAGAAATTCTCGAGTTTCTCTA GTTCAGGCCAGTT-3'
sh-YBX1 #1 F	5'-CCGGCGGCAATGAAGAAGATAAATTCTCGAGTTTATCTTCTTCAT TGCCGTTTTTTTG-3'
sh-YBX1 #1 R	5'-AATTCAAAAACGGCAATGAAGAAGATAAATTCTCGAGTTTATCTT CTTCATTGCCGTT-3'
sh-YBX1 #2 F	5'-CCGGCTGCCATAAAGAAGAATAATTCTCGAGTTATTCTTCTTTAT GGCAGTTTTTTTG-3'
sh-YBX1 #2 R	5'-AATTCAAAAACTGCCATAAAGAAGAATAATTCTCGAGTTATTCTT CTTTATGGCAGTT-3'
sh-MYC #1 F	5'-CCGGCCACCCATAAATCAATAAATTCTCGAGTTTATTGATTTATG GGTGGTTTTTTTG-3'
sh-MYC #1 R	5'-AATTCAAAAACCACCCATAAATCAATAAATTCTCGAGTTTATTGA TTTATGGGTGGTT-3'
sh-MYC #2 F	5'-CCGGGAGGATATCTGGAAGAAATTTCTCGAGATTTCTTCCAGAT ATCCTCTTTTTTTG-3'
sh-MYC #2 R	5'-AATTCAAAAAGAGGATATCTGGAAGAAATTTCTCGAGATTTCTT CCAGATATCCTCTT-3'

Table S4. Primer pairs for luciferase vector construction.

Primer set	Sequence	Location
IL1R1 (F)	5'-CGGGGTACCTATTTCTCAAGTTACCCAGGCACA-3'	–424 – + 87
IL1R1 (R)	5'-CCGCTCGAGGTCGAGACAAATGCCTTGGA-3'	
RAB27B (F)	5'-CGGGGTACCACAGCGCCCTGGGCTCTTTG-3'	–280 – + 241
RAB27B (R)	5'-CCGCTCGAGCCGCCTTCCCAACTCACCAG-3'	

Table S5. Mass spectrometry analysis of proteins pulled down by GAS6-AS1

ABCF1	FBL	NOP56	SFPQ
ABCF2	FERMT3	PHB2	SRP14
ALDOA	HEXIM1	PIP4K2A	SRP9
AP2M1	HSP90B1	PIP4K2C	TAOK1
ARRB2	HSPA5	PLEC	TAOK2
BMS1	HSPA8	PRPF40A	TAOK3
BTK	HSPB1	PRSS3P2	TCP1
CFL1	HSPD1	RAB11A	TRMT6
CMAS	LDHA	RPL18A	TRMT61A
CSNK2A1	LDHB	RPL23	VANGL1
CSNK2B	LMNA	RPL3	YBX1
CTSG	MCM7	RPL32	
DDX21	MSN	RPL35A	
DDX3Y	MTHFD1	RPL36	
DKC1	MYO1G	RPL7	
DRG1	NACA	RPL9	
DSG1	NCL	RPS23	
DSP	NHP2	RPS3A	
EIF1AX	NONO	RPS9	
EIF2S3	NOP10	SEPTIN7	

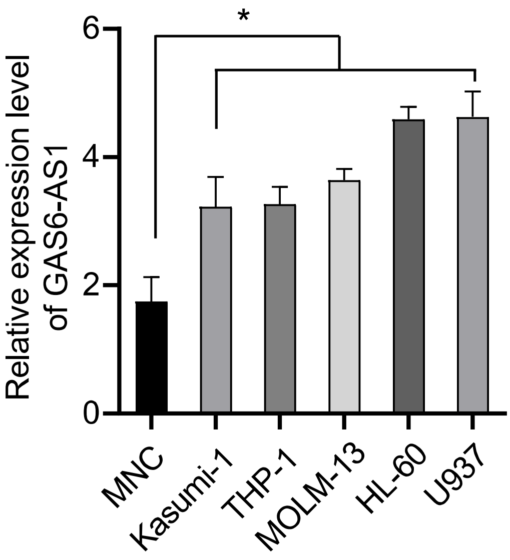

Figure S1. Real-time qRT-PCR (normalized to β-actin, n = 4) showing the expression of GAS6-AS1 in AML cell lines and mononuclear cells (MNC) from healthy donors. Data are shown as mean ± s.e.m. (error bars). **P* < 0.05.

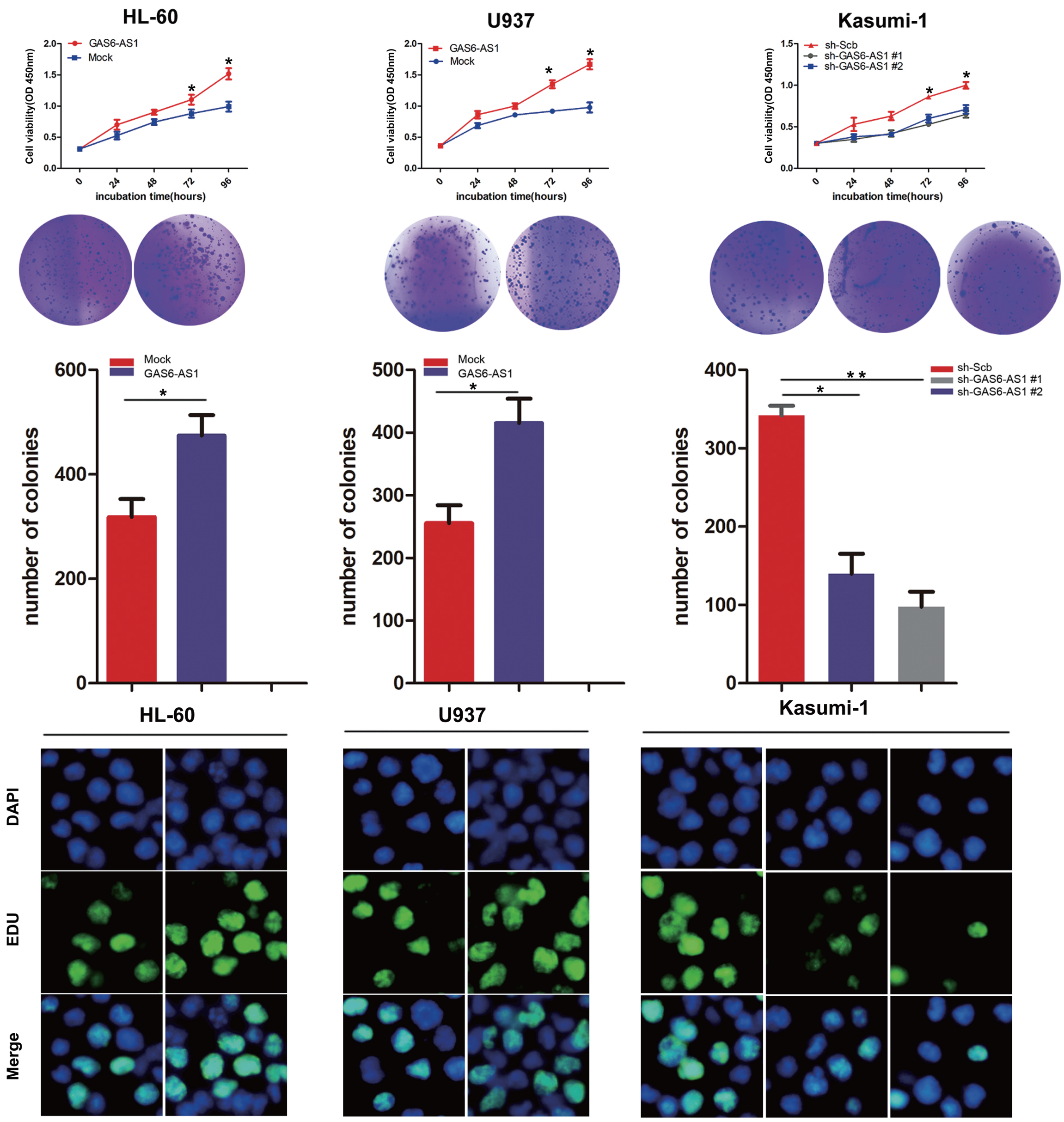

Figure S2. GAS6-AS1 promotes leukemia cell proliferation in vitro and vivo. (A) CCK-8 assays detecting cell proliferation of HL-60, U937 and Kasumi-1 cells following transfection-mediated GAS6-AS1 overexpression or knockdown. (B-C) Colony-forming assays detecting cell proliferation of HL-60, U937 and Kasumi-1 cells. (D) EdU assays examining cell proliferation after transfection. Data were depicted as mean ± s.e.m., *$P < 0.05$.

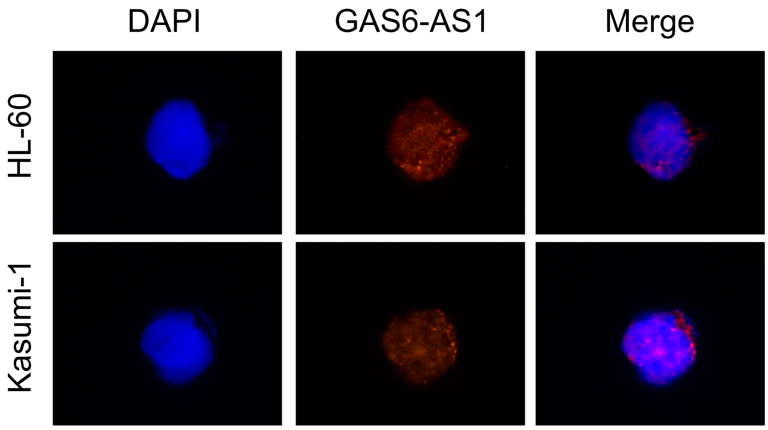

Figure S3. FISH analysis of GAS6-AS1 in HL-60 and Kasumi-1 cells using a biotin-labeled RNA probe. Nuclei were stained with DAPI.

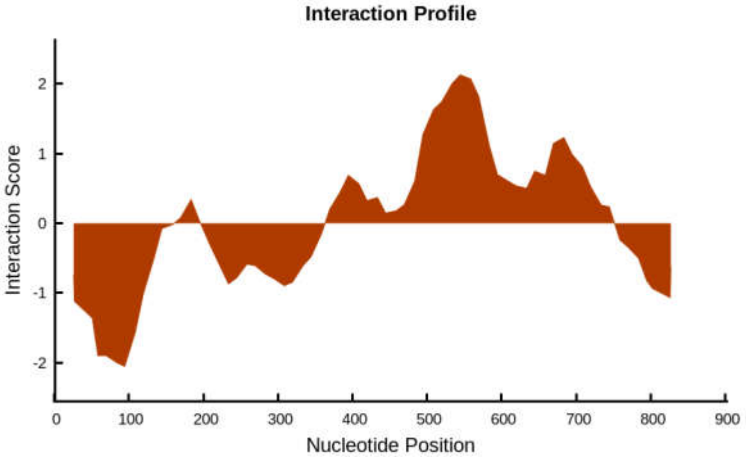

#	Protein region	RNA region	Interaction Propensity	Discriminative Power	Normalized Score
1	249-300	526-577	16.34	45	3.42
2	249-300	527-578	16.04	45	3.34
3	249-300	677-728	15.07	42	3.10
4	174-225	526-577	14.42	40	2.93
5	174-225	677-728	14.33	40	2.91
6	174-225	527-578	14.16	40	2.87
7	249-300	576-627	13.62	37	2.73
8	249-300	577-628	13.19	37	2.62
9	126-177	526-577	13.12	37	2.60
10	249-300	152-203	12.88	35	2.54
11	126-177	527-578	12.85	35	2.54
12	249-300	401-452	12.83	35	2.53
13	74-125	526-577	12.65	35	2.48
14	174-225	577-628	12.57	35	2.46
15	249-300	676-727	12.46	35	2.44
16	126-177	677-728	12.43	35	2.43
17	74-125	527-578	12.37	35	2.41
18	174-225	576-627	12.31	35	2.40
19	174-225	552-603	12.31	35	2.40
20	249-300	652-703	12.29	35	2.39

Figure S4. GAS6-AS1 physically interacts with YBX1 protein. The overall interaction propensityof GAS6-AS1 and YBX1 protein was predicted by catRAPID.

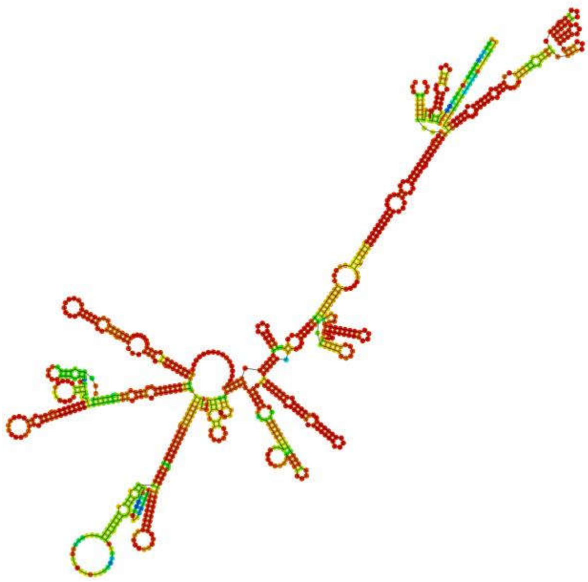

Figure S5. The secondary structure of GAS6-AS1 predicted by RNAfold webserver.

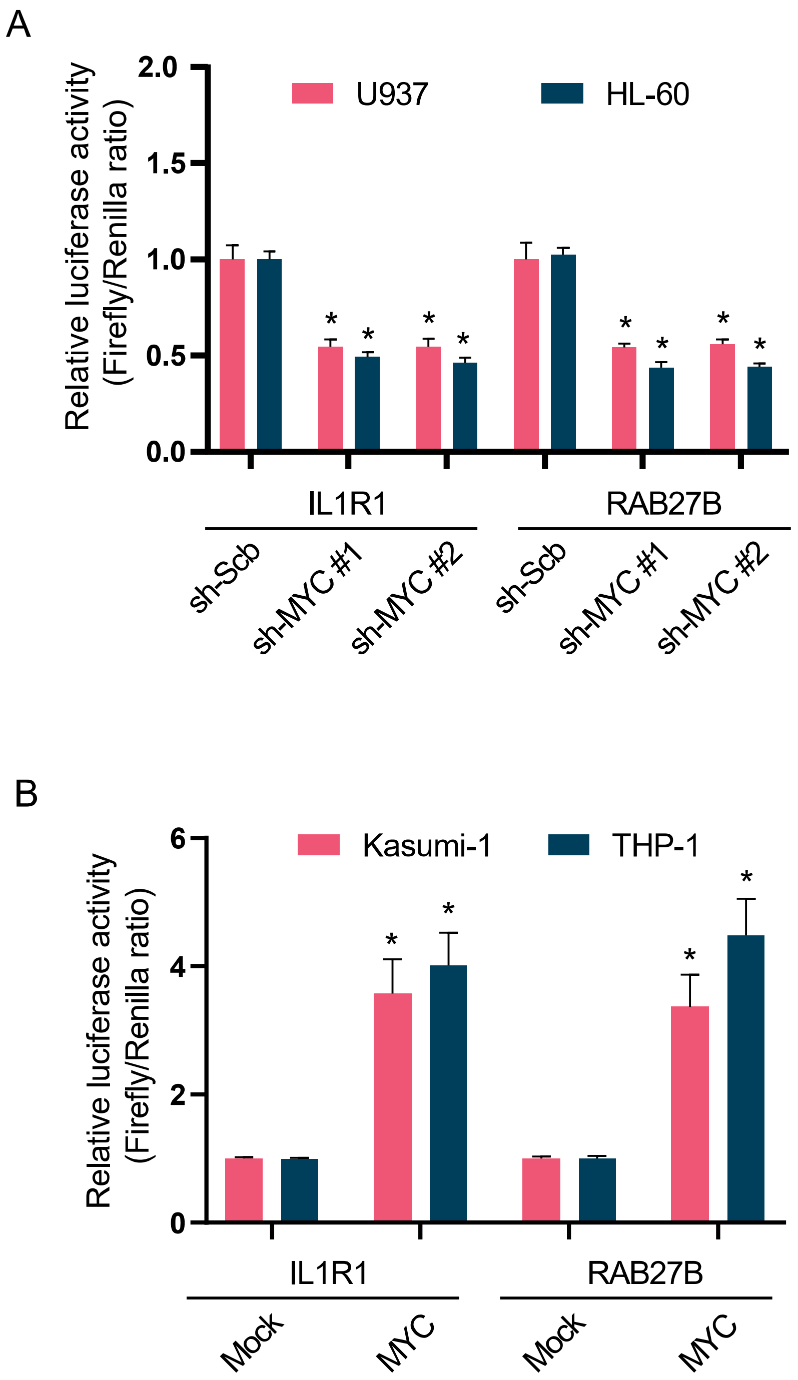

Figure S6. Dual luciferase reporter assays demonstrating the MYC enrichment and promoter activity of IL1R1 and RAB27B in AML cells stably transfected with sh-Scb, sh-MYC #1, sh-MYC #2, mock, or MYC (n = 4). Data are shown as mean ± s.e.m. (error bars). *$P < 0.05$.

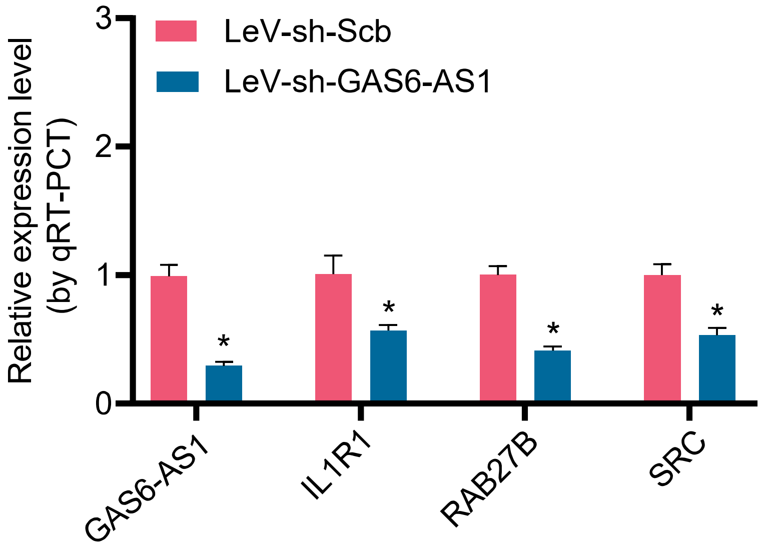

Figure S7. Real-time qRT-PCR (normalized to β-actin) indicating the expression of GAS6-AS1 and its target genes in xenograft tumors established by subcutaneous implantation of U937 cells into both dorsal flanks of each NOD-SCID mouse (n=5 per group) that received intratumoral injection of LeV-sh-Scb or LeV-sh-GAS6-AS1 #1. Data are shown as mean ± s.e.m. (error bars). *$P < 0.05$.
